# Bis(2-hy­droxy­eth­yl)ammonium picrate

**DOI:** 10.1107/S1600536813021697

**Published:** 2013-08-21

**Authors:** Perumal Nagapandiselvi, Rengasamy Gopalakrishnan

**Affiliations:** a Department of Physics, Anna University, Chennai 600 025, India

## Abstract

The asymmetric unit of the title salt, C_4_H_12_NO_2_^+^·C_6_H_2_N_3_O_7_^−^, contain two bis­(2-hy­droxy­eth­yl)ammonium cations and two picrate anions. An intra­molecular N—H⋯O hydrogen bond occurs in each cation. In the crystal, mol­ecules are linked *via* O—H⋯O and N—H⋯O hydrogen bonds, which generate two *R*_2_^1^(6), an *R*_2_^2^(10) and an *R*_2_^2^(13) graph-set ring motifs. There are also a number of C—H⋯O hydrogen bonds present. The sum of these inter­actions leads to the formation a three-dimensional structure.

## Related literature

For general background to picrate complexes, see: In *et al.* (1997 ▶); Zaderenko *et al.* 1997 ▶); Ashwell *et al.* (1995 ▶); Owen & White (1976 ▶); Shakir *et al.* (2009 ▶). For graph-set notation, see: Bernstein *et al.* (1995 ▶).
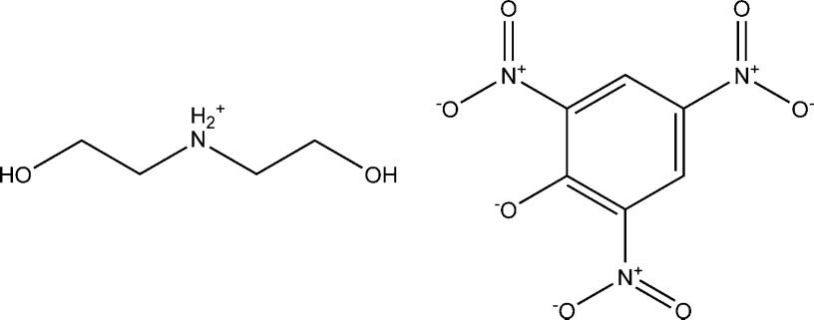


## Experimental

### 

#### Crystal data

C_4_H_12_NO_2_^+^·C_6_H_2_N_3_O_7_^−^
*M* *_r_* = 334.25
Monoclinic, 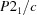

*a* = 24.9396 (6) Å
*b* = 6.9158 (2) Å
*c* = 16.2974 (5) Å
β = 94.608 (1)°
*V* = 2801.85 (14) Å^3^
*Z* = 8
Mo *K*α radiation
μ = 0.14 mm^−1^
*T* = 293 K
0.35 × 0.30 × 0.25 mm

#### Data collection

Bruker SMART APEXII area-detector diffractometer
Absorption correction: multi-scan (*SADABS*; Bruker, 2008 ▶) *T*_min_ = 0.882, *T*_max_ = 0.966
31581 measured reflections
7194 independent reflections
5200 reflections with *I* > 2σ(*I*)
*R*_int_ = 0.032

#### Refinement

*R*[*F* ^2^ > 2σ(*F* ^2^)] = 0.045
*wR*(*F* ^2^) = 0.126
*S* = 1.03
7194 reflections
436 parameters
4 restraints
H atoms treated by a mixture of independent and constrained refinement
Δρ_max_ = 0.52 e Å^−3^
Δρ_min_ = −0.33 e Å^−3^

### 

Data collection: *APEX2* (Bruker, 2008 ▶); cell refinement: *SAINT* (Bruker, 2008 ▶); data reduction: *SAINT*; program(s) used to solve structure: *SHELXS97* (Sheldrick, 2008 ▶); program(s) used to refine structure: *SHELXL97* (Sheldrick, 2008 ▶); molecular graphics: *ORTEP-3 for Windows* (Farrugia, 2012 ▶); software used to prepare material for publication: *SHELXL97* and *PLATON* (Spek, 2009 ▶).

## Supplementary Material

Crystal structure: contains datablock(s) global, I. DOI: 10.1107/S1600536813021697/su2621sup1.cif

Structure factors: contains datablock(s) I. DOI: 10.1107/S1600536813021697/su2621Isup2.hkl

Supplementary material file. DOI: 10.1107/S1600536813021697/su2621Isup3.cml

Additional supplementary materials:  crystallographic information; 3D view; checkCIF report

## Figures and Tables

**Table 1:** Hydrogen-bond geometry (Å, °)

*D*—H⋯*A*	*D*—H	H⋯*A*	*D*⋯*A*	*D*—H⋯*A*
N7—H7*A*⋯O15	0.91 (1)	2.48 (2)	2.8235 (19)	103 (1)
N7—H7*A*⋯O17^i^	0.91 (1)	2.43 (2)	2.9853 (18)	120 (1)
N7—H7*A*⋯O18^ii^	0.91 (1)	2.21 (1)	2.9272 (18)	136 (2)
N7—H7*B*⋯O8^i^	0.91 (2)	1.97 (2)	2.8359 (18)	157 (2)
N7—H7*B*⋯O14^i^	0.91 (2)	2.36 (2)	2.969 (2)	125 (1)
N8—H8*A*⋯O15^iii^	0.91 (2)	2.05 (2)	2.9076 (18)	157 (1)
N8—H8*B*⋯O18	0.91 (2)	2.56 (2)	2.898 (2)	103 (1)
N8—H8*B*⋯O16^iv^	0.91 (2)	1.96 (2)	2.8523 (18)	170 (2)
O15—H15⋯O1	0.82	2.12	2.7891 (16)	139
O15—H15⋯O2	0.82	2.41	3.138 (2)	149
O16—H16⋯O17^i^	0.82	2.24	2.9822 (18)	150
O17—H17⋯O8	0.82	2.00	2.7323 (14)	148
O17—H17⋯O9	0.82	2.27	2.9079 (19)	134
O18—H18⋯O1^iv^	0.82	1.96	2.7453 (18)	161
C3—H3⋯O7^iv^	0.93	2.59	3.502 (2)	167
C9—H9⋯O13^i^	0.93	2.50	3.425 (2)	176
C17—H17*A*⋯O12^v^	0.97	2.50	3.359 (2)	147
C19—H19*A*⋯O7^iii^	0.97	2.45	3.319 (3)	148
C19—H19*B*⋯O5^vi^	0.97	2.44	3.224 (2)	138
C21—H21*B*⋯O1^iii^	0.97	2.33	3.195 (2)	148

Symmetry codes: (i) 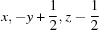; (ii) 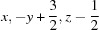; (iii) 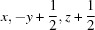; (iv) 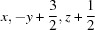; (v) 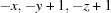; (vi) 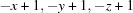.

**Fig. 1.**
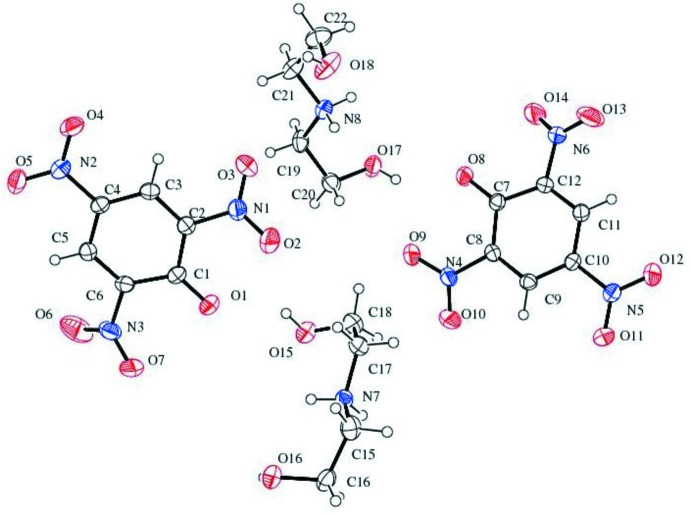
The molecular structure of the title compound, with atom labelling. Displacement ellipsoids are drawn at the 30% probability level.

**Fig. 2.**
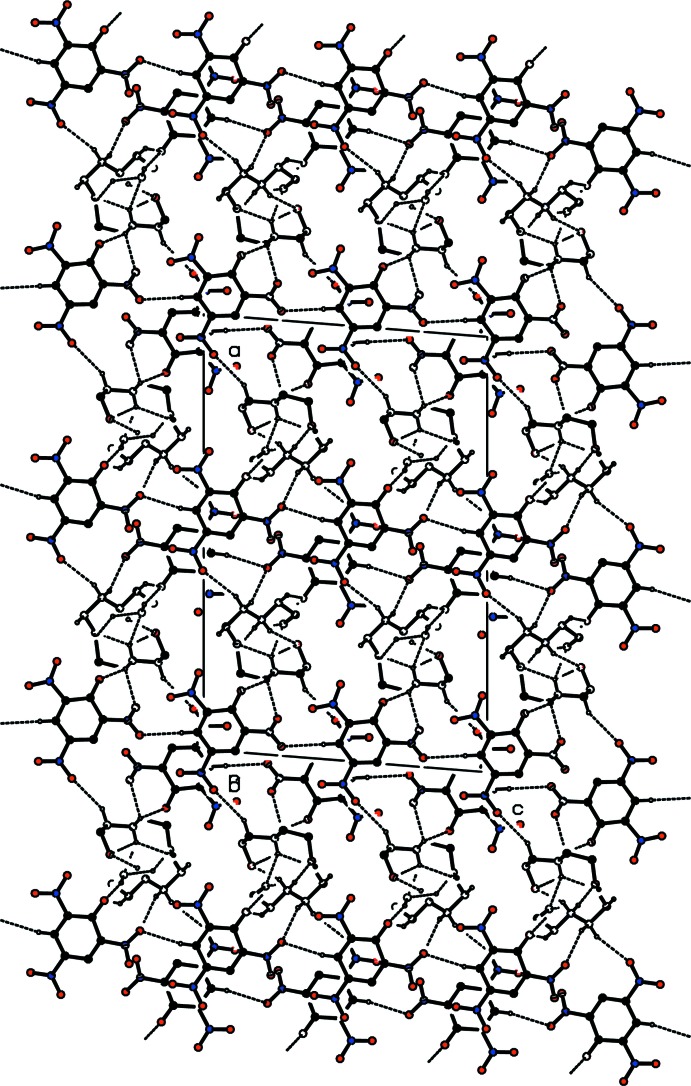
The crystal packing of the title compound viewed along the b axis. Hydrogen bonds are shown as dashed lines (see Table 1 for details). H-atoms not involved in hydrogen bonding have been omitted for clarity.

## supplementary crystallographic information

### 1. Comment

It is well known that picric acid forms charge transfer molecular complexes with a number of aromatic compounds such as aromatic hydrocarbons and amines, through electrostatic or hydrogen bonding interactions (In *et al.*, 1997; Zaderenko *et al.*, 1997). The bonding of donor-acceptor picric acid complexes strongly depends on the nature of partners. Some of the picric acid complexes crystallize in centrosymmetric space groups but have non-linear optical properties (NLO) [Shakir *et al.*, 2009]. This is due to the aggregation of the donor-acceptor molecules in a non-centrosymmetric manner which contributes to the bulk susceptibility from intermolecular charge transfer (Ashwell *et al.*, 1995; Owen & White, 1976). We report herein on the crystal structure of the title salt.

The asymmetric unit of the title salt, Fig. 1, contains two picrate anions and two bis(2-hydroxyethyl)ammonium cations. The amine molecule exists as ammonium ion due to protonation. The picric acid exists as a picrate anion since the proton is transferred to the amine.

The picrate benzene rings (C1-C6 and C7-C12) are inclined to one another by 39.47 (7) °.

In the crystal, molecules are linked via O—H···O and N—H···O hydrogen bonds, which generate two R_2_^1^(6), an R_2_^2^(10) and an R_2_^2^(13) graph-set ring motifs (Bernstein *et al.*, 1995), forming a three-dimensional structure (Table 1 and Fig. 2). There are also C-H···O hydrogen bonds present (Table 1).

### 2. Experimental

An equimolar mixture of 2,2'-azanediylbis(ethan-1-ol) (1.05 mmol) and picric acid (2.29 mmol) in an ethanol solution was stirred over 4 h to attain a saturated homogeneous mixture. The light yellow coloured solution turned a dark yellow and product formation was confirmed using TLC. The saturated solution was filtered into a clean beaker and kept in a constant temperature bath at 303 K. Yellow coloured prism-like crystals suitable for X-ray diffraction analysis were harvested in 2 days.

### 3. Refinement

The N-bound H atoms were located in a difference Fourier map and refined with distance restraints: N-H = 0.91 (1) Å. C-bound H atoms were positioned geometrically (C–H = 0.93 - 0.97 Å) and allowed to ride on their parent atom, with U_iso_(H) = 1.5U_eq_(C) for methyl H atoms and = 1.2U_eq_(C) for other H atoms.

### Crystal data

**Table:**

C_4_H_12_NO_2_^+^·C_6_H_2_N_3_O_7_^−^	*F*(000) = 1392
*M**_r_* = 334.25	*D*_x_ = 1.585 Mg m^−^^3^
Monoclinic, *P*2_1_/*c*	Mo *K*α radiation, λ = 0.71073 Å
Hall symbol: -P 2ybc	Cell parameters from 8443 reflections
*a* = 24.9396 (6) Å	θ = 2.5–28.2°
*b* = 6.9158 (2) Å	µ = 0.14 mm^−^^1^
*c* = 16.2974 (5) Å	*T* = 293 K
β = 94.608 (1)°	Block, yellow
*V* = 2801.85 (14) Å^3^	0.35 × 0.30 × 0.25 mm
*Z* = 8	

### Data collection

**Table:**

Bruker SMART APEXII area-detector diffractometer	7194 independent reflections
Radiation source: fine-focus sealed tube	5200 reflections with *I* > 2σ(*I*)
Graphite monochromator	*R*_int_ = 0.032
ω and φ scans	θ_max_ = 28.7°, θ_min_ = 2.5°
Absorption correction: multi-scan (*SADABS*; Bruker, 2008)	*h* = −33→33
*T*_min_ = 0.882, *T*_max_ = 0.966	*k* = −8→9
31581 measured reflections	*l* = −21→20

### Refinement

**Table:**

Refinement on *F*^2^	Secondary atom site location: difference Fourier map
Least-squares matrix: full	Hydrogen site location: inferred from neighbouring sites
*R*[*F*^2^ > 2σ(*F*^2^)] = 0.045	H atoms treated by a mixture of independent and constrained refinement
*wR*(*F*^2^) = 0.126	*w* = 1/[σ^2^(*F*_o_^2^) + (0.0578*P*)^2^ + 0.8326*P*] where *P* = (*F*_o_^2^ + 2*F*_c_^2^)/3
*S* = 1.03	(Δ/σ)_max_ < 0.001
7194 reflections	Δρ_max_ = 0.52 e Å^−^^3^
436 parameters	Δρ_min_ = −0.33 e Å^−^^3^
4 restraints	Extinction correction: *SHELXL97* (Sheldrick, 2008), Fc^*^=kFc[1+0.001xFc^2^λ^3^/sin(2θ)]^-1/4^
Primary atom site location: structure-invariant direct methods	Extinction coefficient: 0.0026 (5)

### Special details

**Table:**

Geometry. Bond distances, angles etc. have been calculated using the rounded fractional coordinates. All su's are estimated from the variances of the (full) variance-covariance matrix. The cell esds are taken into account in the estimation of distances, angles and torsion angles
Refinement. Refinement of F^2^ against ALL reflections. The weighted R-factor wR and goodness of fit S are based on F^2^, conventional R-factors R are based on F, with F set to zero for negative F^2^. The threshold expression of F^2^ > 2sigma(F^2^) is used only for calculating R-factors(gt) etc. and is not relevant to the choice of reflections for refinement. R-factors based on F^2^ are statistically about twice as large as those based on F, and R- factors based on ALL data will be even larger.

### Fractional atomic coordinates and isotropic or equivalent isotropic displacement parameters (Å^2^)

**Table:**

	*x*	*y*	*z*	*U*_iso_*/*U*_eq_	
O1	0.36588 (4)	0.55080 (17)	0.35068 (7)	0.0376 (4)	
O2	0.31475 (5)	0.6612 (3)	0.48109 (9)	0.0732 (6)	
O3	0.36173 (6)	0.6189 (3)	0.59420 (9)	0.0770 (7)	
O4	0.54463 (5)	0.8458 (2)	0.60986 (8)	0.0524 (5)	
O5	0.58962 (5)	0.8254 (2)	0.50363 (8)	0.0539 (5)	
O6	0.50603 (8)	0.4989 (3)	0.26298 (10)	0.0854 (7)	
O7	0.42846 (7)	0.6218 (3)	0.22486 (8)	0.0879 (8)	
N1	0.35824 (6)	0.6443 (2)	0.52065 (9)	0.0414 (5)	
N2	0.54771 (5)	0.81045 (19)	0.53708 (8)	0.0343 (4)	
N3	0.46430 (7)	0.5815 (3)	0.27694 (9)	0.0492 (5)	
C1	0.40616 (6)	0.6139 (2)	0.39305 (9)	0.0286 (4)	
C2	0.40723 (6)	0.6641 (2)	0.47915 (9)	0.0301 (4)	
C3	0.45221 (6)	0.7261 (2)	0.52603 (9)	0.0298 (4)	
C4	0.49989 (6)	0.7465 (2)	0.48898 (9)	0.0287 (4)	
C5	0.50313 (6)	0.6991 (2)	0.40681 (9)	0.0314 (4)	
C6	0.45820 (6)	0.6370 (2)	0.36189 (9)	0.0311 (4)	
O8	0.14146 (4)	0.04467 (19)	0.63223 (7)	0.0408 (4)	
O9	0.17482 (5)	0.1133 (3)	0.48253 (9)	0.0708 (6)	
O10	0.11402 (5)	0.0955 (2)	0.38420 (8)	0.0597 (5)	
O11	−0.05810 (5)	0.3536 (2)	0.41733 (7)	0.0477 (4)	
O12	−0.09095 (5)	0.3430 (2)	0.53515 (8)	0.0551 (5)	
O13	0.02107 (7)	0.1656 (3)	0.77418 (9)	0.0919 (8)	
O14	0.08393 (6)	−0.0338 (3)	0.76020 (9)	0.0722 (6)	
N4	0.12787 (5)	0.1159 (2)	0.45685 (8)	0.0371 (4)	
N5	−0.05404 (5)	0.3196 (2)	0.49110 (8)	0.0335 (4)	
N6	0.05233 (6)	0.0868 (3)	0.73232 (8)	0.0467 (5)	
C7	0.09722 (6)	0.1020 (2)	0.59992 (9)	0.0286 (4)	
C8	0.08651 (6)	0.1477 (2)	0.51350 (9)	0.0280 (4)	
C9	0.03840 (6)	0.2168 (2)	0.47875 (9)	0.0286 (4)	
C10	−0.00312 (6)	0.2474 (2)	0.52799 (9)	0.0282 (4)	
C11	0.00245 (6)	0.2070 (2)	0.61143 (9)	0.0321 (4)	
C12	0.05015 (6)	0.1339 (2)	0.64497 (9)	0.0316 (4)	
O15	0.25842 (5)	0.43596 (19)	0.33076 (8)	0.0432 (4)	
O16	0.23692 (4)	0.95252 (18)	0.11889 (8)	0.0398 (4)	
N7	0.19095 (5)	0.7158 (2)	0.24951 (8)	0.0332 (4)	
C15	0.17130 (7)	0.9075 (3)	0.21928 (11)	0.0423 (6)	
C16	0.18137 (6)	0.9372 (3)	0.13031 (11)	0.0394 (5)	
C17	0.19148 (7)	0.6884 (3)	0.34013 (10)	0.0407 (5)	
C18	0.20910 (7)	0.4870 (3)	0.36335 (11)	0.0456 (6)	
O17	0.24822 (4)	−0.02326 (19)	0.61782 (8)	0.0424 (4)	
O18	0.30269 (5)	0.6577 (2)	0.78140 (10)	0.0560 (5)	
N8	0.30854 (5)	0.2811 (2)	0.70463 (8)	0.0318 (4)	
C19	0.32980 (6)	0.1541 (3)	0.64123 (11)	0.0396 (5)	
C20	0.28589 (7)	0.0922 (3)	0.57860 (10)	0.0396 (5)	
C21	0.35099 (7)	0.3627 (3)	0.76446 (11)	0.0409 (5)	
C22	0.32629 (8)	0.4956 (3)	0.82332 (12)	0.0505 (6)	
H3	0.45060	0.75380	0.58160	0.0360*	
H5	0.53560	0.70980	0.38280	0.0380*	
H9	0.03390	0.24270	0.42260	0.0340*	
H11	−0.02580	0.22930	0.64410	0.0390*	
H7A	0.2259 (4)	0.703 (3)	0.2383 (12)	0.048 (5)*	
H7B	0.1700 (6)	0.623 (2)	0.2236 (11)	0.045 (5)*	
H15	0.28350	0.48610	0.35860	0.0650*	
H15A	0.18940	1.00830	0.25240	0.0510*	
H15B	0.13300	0.91760	0.22540	0.0510*	
H16	0.24850	0.84640	0.10650	0.0600*	
H16A	0.16630	0.82940	0.09810	0.0470*	
H16B	0.16320	1.05400	0.11020	0.0470*	
H17A	0.15580	0.71130	0.35760	0.0490*	
H17B	0.21590	0.78100	0.36800	0.0490*	
H18A	0.21330	0.47650	0.42290	0.0550*	
H18B	0.18140	0.39650	0.34320	0.0550*	
H8A	0.2849 (6)	0.214 (2)	0.7332 (10)	0.041 (5)*	
H8B	0.2887 (7)	0.377 (2)	0.6794 (11)	0.045 (5)*	
H17	0.21760	0.01330	0.60360	0.0640*	
H18	0.32190	0.75280	0.79060	0.0840*	
H19A	0.34630	0.04070	0.66760	0.0480*	
H19B	0.35720	0.22300	0.61400	0.0480*	
H20A	0.26800	0.20500	0.55390	0.0470*	
H20B	0.30100	0.01840	0.53540	0.0470*	
H21A	0.37710	0.43310	0.73500	0.0490*	
H21B	0.36960	0.25850	0.79470	0.0490*	
H22A	0.29900	0.42650	0.85090	0.0610*	
H22B	0.35370	0.53900	0.86480	0.0610*	

### Atomic displacement parameters (Å^2^)

**Table:**

	*U*^11^	*U*^22^	*U*^33^	*U*^12^	*U*^13^	*U*^23^
O1	0.0327 (6)	0.0383 (7)	0.0405 (6)	−0.0014 (5)	−0.0049 (5)	−0.0058 (5)
O2	0.0330 (7)	0.1269 (15)	0.0599 (9)	0.0037 (8)	0.0055 (6)	−0.0091 (9)
O3	0.0550 (9)	0.1382 (16)	0.0394 (8)	−0.0213 (10)	0.0137 (6)	0.0033 (9)
O4	0.0448 (7)	0.0731 (10)	0.0374 (7)	−0.0020 (7)	−0.0075 (5)	−0.0136 (6)
O5	0.0322 (6)	0.0742 (10)	0.0553 (8)	−0.0116 (6)	0.0039 (5)	−0.0109 (7)
O6	0.1145 (14)	0.0906 (13)	0.0560 (10)	0.0195 (12)	0.0371 (10)	−0.0135 (9)
O7	0.0681 (10)	0.1635 (19)	0.0303 (7)	−0.0470 (11)	−0.0073 (7)	0.0040 (9)
N1	0.0341 (7)	0.0495 (9)	0.0412 (8)	−0.0036 (6)	0.0071 (6)	−0.0049 (7)
N2	0.0324 (7)	0.0306 (7)	0.0388 (7)	0.0015 (5)	−0.0041 (5)	−0.0019 (6)
N3	0.0614 (10)	0.0577 (10)	0.0291 (7)	−0.0219 (8)	0.0074 (7)	−0.0032 (7)
C1	0.0299 (7)	0.0227 (7)	0.0322 (7)	0.0029 (6)	−0.0031 (6)	0.0021 (6)
C2	0.0301 (7)	0.0285 (8)	0.0319 (7)	0.0031 (6)	0.0032 (6)	0.0016 (6)
C3	0.0351 (8)	0.0270 (8)	0.0269 (7)	0.0037 (6)	0.0005 (6)	0.0002 (6)
C4	0.0305 (7)	0.0251 (8)	0.0298 (7)	0.0018 (6)	−0.0026 (6)	0.0016 (6)
C5	0.0303 (7)	0.0315 (8)	0.0326 (8)	−0.0014 (6)	0.0041 (6)	0.0011 (6)
C6	0.0370 (8)	0.0310 (8)	0.0251 (7)	−0.0019 (6)	0.0015 (6)	0.0014 (6)
O8	0.0284 (5)	0.0552 (8)	0.0391 (6)	0.0067 (5)	0.0040 (4)	0.0128 (5)
O9	0.0300 (7)	0.1358 (16)	0.0478 (8)	0.0092 (8)	0.0099 (6)	0.0189 (9)
O10	0.0523 (8)	0.0944 (12)	0.0335 (7)	0.0120 (8)	0.0109 (6)	−0.0077 (7)
O11	0.0392 (6)	0.0665 (9)	0.0364 (7)	0.0055 (6)	−0.0039 (5)	0.0083 (6)
O12	0.0319 (6)	0.0845 (11)	0.0499 (8)	0.0147 (7)	0.0087 (5)	0.0086 (7)
O13	0.0803 (11)	0.164 (2)	0.0331 (7)	0.0529 (12)	0.0147 (7)	0.0004 (10)
O14	0.0509 (8)	0.1207 (15)	0.0461 (8)	0.0241 (9)	0.0109 (6)	0.0377 (9)
N4	0.0325 (7)	0.0452 (8)	0.0347 (7)	0.0036 (6)	0.0090 (5)	0.0046 (6)
N5	0.0285 (6)	0.0351 (8)	0.0362 (7)	−0.0009 (5)	−0.0008 (5)	0.0006 (6)
N6	0.0336 (7)	0.0782 (12)	0.0285 (7)	0.0044 (8)	0.0030 (6)	0.0045 (7)
C7	0.0268 (7)	0.0283 (8)	0.0306 (7)	−0.0015 (6)	0.0019 (5)	0.0008 (6)
C8	0.0274 (7)	0.0281 (8)	0.0292 (7)	−0.0020 (6)	0.0063 (5)	−0.0004 (6)
C9	0.0311 (7)	0.0284 (8)	0.0261 (7)	−0.0036 (6)	0.0014 (5)	−0.0004 (6)
C10	0.0252 (7)	0.0285 (8)	0.0304 (7)	−0.0011 (6)	−0.0003 (5)	−0.0015 (6)
C11	0.0272 (7)	0.0384 (9)	0.0309 (7)	−0.0013 (6)	0.0037 (6)	−0.0041 (6)
C12	0.0310 (7)	0.0390 (9)	0.0249 (7)	−0.0014 (6)	0.0025 (6)	−0.0009 (6)
O15	0.0363 (6)	0.0448 (7)	0.0475 (7)	−0.0010 (5)	−0.0023 (5)	−0.0025 (6)
O16	0.0305 (6)	0.0399 (7)	0.0488 (7)	−0.0030 (5)	0.0021 (5)	−0.0031 (6)
N7	0.0286 (6)	0.0364 (8)	0.0349 (7)	0.0015 (6)	0.0044 (5)	−0.0043 (6)
C15	0.0371 (9)	0.0378 (10)	0.0528 (10)	0.0087 (7)	0.0083 (7)	−0.0007 (8)
C16	0.0281 (8)	0.0405 (10)	0.0484 (10)	0.0033 (7)	−0.0036 (7)	0.0052 (8)
C17	0.0384 (9)	0.0521 (11)	0.0324 (8)	−0.0010 (8)	0.0077 (7)	−0.0044 (7)
C18	0.0436 (10)	0.0525 (11)	0.0413 (9)	−0.0077 (8)	0.0078 (7)	0.0075 (8)
O17	0.0297 (6)	0.0469 (7)	0.0504 (7)	−0.0026 (5)	0.0015 (5)	0.0081 (6)
O18	0.0441 (7)	0.0416 (8)	0.0780 (10)	0.0070 (6)	−0.0221 (7)	−0.0157 (7)
N8	0.0267 (6)	0.0302 (7)	0.0381 (7)	0.0011 (5)	−0.0001 (5)	0.0000 (6)
C19	0.0273 (7)	0.0413 (10)	0.0512 (10)	0.0021 (7)	0.0092 (7)	−0.0068 (8)
C20	0.0393 (9)	0.0429 (10)	0.0374 (9)	−0.0037 (7)	0.0086 (7)	−0.0053 (7)
C21	0.0363 (8)	0.0320 (9)	0.0515 (10)	0.0001 (7)	−0.0134 (7)	0.0015 (7)
C22	0.0609 (12)	0.0408 (11)	0.0476 (10)	−0.0075 (9)	−0.0098 (9)	−0.0069 (8)

### Geometric parameters (Å, º)

**Table:**

O1—C1	1.2510 (18)	C1—C2	1.444 (2)
O2—N1	1.222 (2)	C1—C6	1.440 (2)
O3—N1	1.208 (2)	C2—C3	1.374 (2)
O4—N2	1.2195 (18)	C3—C4	1.383 (2)
O5—N2	1.2214 (18)	C4—C5	1.387 (2)
O6—N3	1.224 (3)	C5—C6	1.358 (2)
O7—N3	1.215 (2)	C3—H3	0.9300
O8—C7	1.2484 (18)	C5—H5	0.9300
O9—N4	1.2115 (18)	C7—C8	1.447 (2)
O10—N4	1.2147 (18)	C7—C12	1.451 (2)
O11—N5	1.2212 (17)	C8—C9	1.371 (2)
O12—N5	1.2228 (18)	C9—C10	1.376 (2)
O13—N6	1.207 (2)	C10—C11	1.384 (2)
O14—N6	1.211 (3)	C11—C12	1.366 (2)
O15—C18	1.423 (2)	C9—H9	0.9300
O16—C16	1.4166 (18)	C11—H11	0.9300
O15—H15	0.8200	C15—C16	1.505 (3)
O16—H16	0.8200	C17—C18	1.500 (3)
O17—C20	1.423 (2)	C15—H15A	0.9700
O18—C22	1.416 (2)	C15—H15B	0.9700
O17—H17	0.8200	C16—H16A	0.9700
O18—H18	0.8200	C16—H16B	0.9700
N1—C2	1.450 (2)	C17—H17B	0.9700
N2—C4	1.443 (2)	C17—H17A	0.9700
N3—C6	1.456 (2)	C18—H18A	0.9700
N4—C8	1.455 (2)	C18—H18B	0.9700
N5—C10	1.449 (2)	C19—C20	1.498 (2)
N6—C12	1.457 (2)	C21—C22	1.497 (3)
N7—C15	1.484 (2)	C19—H19A	0.9700
N7—C17	1.488 (2)	C19—H19B	0.9700
N7—H7A	0.909 (11)	C20—H20A	0.9700
N7—H7B	0.910 (15)	C20—H20B	0.9700
N8—C21	1.491 (2)	C21—H21A	0.9700
N8—C19	1.486 (2)	C21—H21B	0.9700
N8—H8B	0.906 (16)	C22—H22A	0.9700
N8—H8A	0.908 (15)	C22—H22B	0.9700
			
C18—O15—H15	109.00	N5—C10—C9	119.09 (13)
C16—O16—H16	109.00	C9—C10—C11	121.39 (14)
C20—O17—H17	109.00	N5—C10—C11	119.50 (13)
C22—O18—H18	109.00	C10—C11—C12	118.90 (14)
O3—N1—C2	118.72 (14)	N6—C12—C7	119.49 (13)
O2—N1—C2	119.37 (14)	N6—C12—C11	115.90 (13)
O2—N1—O3	121.85 (16)	C7—C12—C11	124.61 (13)
O4—N2—C4	118.42 (13)	C10—C9—H9	120.00
O5—N2—C4	118.88 (13)	C8—C9—H9	120.00
O4—N2—O5	122.69 (13)	C12—C11—H11	121.00
O6—N3—O7	124.47 (17)	C10—C11—H11	121.00
O6—N3—C6	117.06 (15)	N7—C15—C16	111.44 (15)
O7—N3—C6	118.47 (17)	O16—C16—C15	112.17 (14)
O9—N4—C8	119.85 (13)	N7—C17—C18	110.35 (15)
O9—N4—O10	121.75 (14)	O15—C18—C17	112.38 (15)
O10—N4—C8	118.39 (12)	C16—C15—H15A	109.00
O11—N5—O12	123.07 (14)	N7—C15—H15B	109.00
O11—N5—C10	118.38 (13)	C16—C15—H15B	109.00
O12—N5—C10	118.55 (13)	H15A—C15—H15B	108.00
O13—N6—O14	121.99 (15)	N7—C15—H15A	109.00
O14—N6—C12	119.52 (15)	O16—C16—H16B	109.00
O13—N6—C12	118.45 (16)	C15—C16—H16A	109.00
C15—N7—C17	114.82 (14)	C15—C16—H16B	109.00
H7A—N7—H7B	111.2 (16)	H16A—C16—H16B	108.00
C17—N7—H7B	109.3 (11)	O16—C16—H16A	109.00
C15—N7—H7B	108.3 (10)	N7—C17—H17A	110.00
C17—N7—H7A	104.8 (12)	N7—C17—H17B	110.00
C15—N7—H7A	108.5 (13)	C18—C17—H17B	110.00
C19—N8—C21	113.83 (12)	H17A—C17—H17B	108.00
H8A—N8—H8B	104.7 (14)	C18—C17—H17A	110.00
C21—N8—H8B	110.7 (10)	C17—C18—H18A	109.00
C19—N8—H8A	109.7 (9)	O15—C18—H18A	109.00
C19—N8—H8B	109.2 (11)	C17—C18—H18B	109.00
C21—N8—H8A	108.3 (10)	H18A—C18—H18B	108.00
O1—C1—C2	124.87 (14)	O15—C18—H18B	109.00
C2—C1—C6	111.67 (13)	N8—C19—C20	111.13 (13)
O1—C1—C6	123.41 (13)	O17—C20—C19	109.09 (14)
C1—C2—C3	124.47 (14)	N8—C21—C22	110.14 (14)
N1—C2—C1	118.70 (13)	O18—C22—C21	110.70 (16)
N1—C2—C3	116.81 (13)	N8—C19—H19A	109.00
C2—C3—C4	118.76 (14)	N8—C19—H19B	109.00
N2—C4—C3	119.74 (13)	C20—C19—H19A	109.00
N2—C4—C5	119.15 (13)	C20—C19—H19B	109.00
C3—C4—C5	121.06 (14)	H19A—C19—H19B	108.00
C4—C5—C6	119.05 (14)	O17—C20—H20A	110.00
N3—C6—C5	116.82 (14)	O17—C20—H20B	110.00
N3—C6—C1	118.16 (13)	C19—C20—H20A	110.00
C1—C6—C5	124.96 (14)	C19—C20—H20B	110.00
C2—C3—H3	121.00	H20A—C20—H20B	108.00
C4—C3—H3	121.00	N8—C21—H21A	110.00
C6—C5—H5	120.00	N8—C21—H21B	110.00
C4—C5—H5	120.00	C22—C21—H21A	110.00
O8—C7—C8	124.58 (14)	C22—C21—H21B	110.00
C8—C7—C12	111.45 (13)	H21A—C21—H21B	108.00
O8—C7—C12	123.96 (13)	O18—C22—H22A	109.00
N4—C8—C9	115.60 (13)	O18—C22—H22B	109.00
C7—C8—C9	124.41 (14)	C21—C22—H22A	110.00
N4—C8—C7	119.97 (13)	C21—C22—H22B	109.00
C8—C9—C10	119.16 (14)	H22A—C22—H22B	108.00
			
O2—N1—C2—C3	152.93 (17)	C6—C1—C2—C3	−0.1 (2)
O2—N1—C2—C1	−28.5 (2)	O1—C1—C6—C5	−177.78 (14)
O3—N1—C2—C1	154.31 (17)	C2—C1—C6—C5	−0.3 (2)
O3—N1—C2—C3	−24.2 (2)	C6—C1—C2—N1	−178.52 (12)
O5—N2—C4—C3	179.11 (14)	C1—C2—C3—C4	1.4 (2)
O4—N2—C4—C3	−0.3 (2)	N1—C2—C3—C4	179.82 (13)
O4—N2—C4—C5	−177.73 (14)	C2—C3—C4—C5	−2.3 (2)
O5—N2—C4—C5	1.6 (2)	C2—C3—C4—N2	−179.68 (13)
O6—N3—C6—C5	37.1 (2)	N2—C4—C5—C6	179.31 (13)
O7—N3—C6—C1	40.8 (3)	C3—C4—C5—C6	1.9 (2)
O7—N3—C6—C5	−141.99 (19)	C4—C5—C6—N3	−177.52 (14)
O6—N3—C6—C1	−140.14 (18)	C4—C5—C6—C1	−0.5 (2)
O9—N4—C8—C7	−24.1 (2)	C8—C7—C12—C11	−3.1 (2)
O9—N4—C8—C9	157.43 (17)	C12—C7—C8—C9	1.4 (2)
O10—N4—C8—C9	−21.5 (2)	O8—C7—C8—N4	4.0 (2)
O10—N4—C8—C7	157.01 (14)	C8—C7—C12—N6	177.03 (14)
O11—N5—C10—C11	−179.74 (14)	O8—C7—C12—N6	−3.8 (2)
O12—N5—C10—C9	178.72 (14)	C12—C7—C8—N4	−176.87 (12)
O12—N5—C10—C11	0.0 (2)	O8—C7—C12—C11	176.07 (15)
O11—N5—C10—C9	−1.0 (2)	O8—C7—C8—C9	−177.71 (15)
O14—N6—C12—C11	155.08 (17)	N4—C8—C9—C10	178.84 (13)
O14—N6—C12—C7	−25.0 (2)	C7—C8—C9—C10	0.5 (2)
O13—N6—C12—C11	−22.5 (2)	C8—C9—C10—N5	−179.71 (13)
O13—N6—C12—C7	157.36 (17)	C8—C9—C10—C11	−1.0 (2)
C17—N7—C15—C16	169.65 (14)	C9—C10—C11—C12	−0.5 (2)
C15—N7—C17—C18	176.94 (14)	N5—C10—C11—C12	178.17 (13)
C21—N8—C19—C20	−174.64 (15)	C10—C11—C12—C7	2.8 (2)
C19—N8—C21—C22	177.33 (15)	C10—C11—C12—N6	−177.37 (14)
C2—C1—C6—N3	176.61 (14)	N7—C15—C16—O16	−67.6 (2)
O1—C1—C2—C3	177.31 (14)	N7—C17—C18—O15	52.13 (19)
O1—C1—C6—N3	−0.8 (2)	N8—C19—C20—O17	−64.2 (2)
O1—C1—C2—N1	−1.1 (2)	N8—C21—C22—O18	−64.1 (2)

### Hydrogen-bond geometry (Å, º)

**Table:**

*D*—H···*A*	*D*—H	H···*A*	*D*···*A*	*D*—H···*A*
N7—H7*A*···O15	0.91 (1)	2.48 (2)	2.8235 (19)	103 (1)
N7—H7*A*···O17^i^	0.91 (1)	2.43 (2)	2.9853 (18)	120 (1)
N7—H7*A*···O18^ii^	0.91 (1)	2.21 (1)	2.9272 (18)	136 (2)
N7—H7*B*···O8^i^	0.91 (2)	1.97 (2)	2.8359 (18)	157 (2)
N7—H7*B*···O14^i^	0.91 (2)	2.36 (2)	2.969 (2)	125 (1)
N8—H8*A*···O15^iii^	0.91 (2)	2.05 (2)	2.9076 (18)	157 (1)
N8—H8*B*···O18	0.91 (2)	2.56 (2)	2.898 (2)	103 (1)
N8—H8*B*···O16^iv^	0.91 (2)	1.96 (2)	2.8523 (18)	170 (2)
O15—H15···O1	0.82	2.12	2.7891 (16)	139
O15—H15···O2	0.82	2.41	3.138 (2)	149
O16—H16···O17^i^	0.82	2.24	2.9822 (18)	150
O17—H17···O8	0.82	2.00	2.7323 (14)	148
O17—H17···O9	0.82	2.27	2.9079 (19)	134
O18—H18···O1^iv^	0.82	1.96	2.7453 (18)	161
C3—H3···O7^iv^	0.93	2.59	3.502 (2)	167
C9—H9···O13^i^	0.93	2.50	3.425 (2)	176
C17—H17*A*···O12^v^	0.97	2.50	3.359 (2)	147
C19—H19*A*···O7^iii^	0.97	2.45	3.319 (3)	148
C19—H19*B*···O5^vi^	0.97	2.44	3.224 (2)	138
C21—H21*B*···O1^iii^	0.97	2.33	3.195 (2)	148

Symmetry codes: (i) *x*, −*y*+1/2, *z*−1/2; (ii) *x*, −*y*+3/2, *z*−1/2; (iii) *x*, −*y*+1/2, *z*+1/2; (iv) *x*, −*y*+3/2, *z*+1/2; (v) −*x*, −*y*+1, −*z*+1; (vi) −*x*+1, −*y*+1, −*z*+1.
